# Association between antibiotic usage during infancy and asthma incidence among children: a population-level ecological study in British Columbia, Canada

**DOI:** 10.3389/falgy.2024.1456077

**Published:** 2024-08-27

**Authors:** Abdullah Al Mamun, Carl Zou, Hannah Lishman, Säde Stenlund, Max Xie, Erica Chuang, David M. Patrick

**Affiliations:** ^1^ School of Population and Public Health, University of British Columbia, Vancouver, BC, Canada; ^2^ British Columbia Centre for Disease Control, Vancouver, BC, Canada

**Keywords:** asthma, antibiotics, breastfeeding, incidence, Canada

## Abstract

**Background:**

This study follows published associations in BC to 2014 (updated in 2019) to model the predicted incidence of asthma in BC children attributable to antibiotic use within the context of reduced antibiotic use and increased breastfeeding in BC infants from 2000 to 2019.

**Methods:**

A population-based ecological study was conducted in BC from 2000 to 2019, using outpatient antibiotic prescription data from BC PharmaNet and asthma diagnoses from the Chronic Disease Registry. Breastfeeding estimates were calculated using the Canadian Community Health Survey (CCHS). Population attributable risk (PAR) was calculated using a blended relative risk (RR) of asthma in antibiotic-exposed children who were and were not breastfed. PAR was used to calculate predicted vs. actual asthma incidence in 2019. Negative binomial regression was used to estimate the association between the average antibiotic prescription rate in infants under 1 and asthma incidence in 1–4 year olds, stratified by periods between 2000–2014 and 2015–2019.

**Results:**

In BC, antibiotic prescribing decreased by 77% in infants under 1 and asthma incidence decreased by 41% in children 1–4 years from 2000 to 2019. BC breastfeeding rates increased from 46% in the 2005 CCHS to 71% in the 2017/18 CCHS. After calculating the PAR using a blended RR, the predicted asthma incidence in 2019 was 18.8/1,000 population. This was comparable to the observed asthma incidence in children 1–4 years of 16.6/1,000 population in 2019. During 2000–2014, adjusted incidence risk ratio (aIRR) for children under Quintile 5 of average antibiotic prescribing was 1.75 (95% CI: 1.63–1.88, *P* < 0.0001) times higher than that for Quintile 1. However, between 2015 and 2019, this association weakened (as expected because of increasing prevalence of breastfeeding), with the expected asthma incidence for Quintile 5 only 11% (aIRR 1.11, 95% CI: 0.78–1.57) higher than for Quintile 1.

**Conclusion:**

We identified that over the past 20 years, antibiotic exposure in infants under 1 and asthma incidence in children 1–4 years has decreased significantly. Decreasing antibiotic exposure and increasing breastfeeding (which further mitigates risk associated with antibiotics) are of sufficient scale to explain much of this population trend. Changes in environmental, social and other exposures remain relevant to this complicated etiological pathway.

## Background/Introduction

A growing body of evidence shows an association between receiving antibiotics during infancy and developing asthma and other atopic diseases in childhood (1). One potential mechanism is disruption of the developing gut microbiota, and impairment of its role in training the developing immune system away from atopy (2). A reduction in gut microbial diversity and the absence of key bacterial taxa has been associated with an increased incidence of atopic disease with evidence continuing to accrue from longitudinal cohort studies (3). Given that antibiotics can significantly affect a child's developing microbiome (4), they have been investigated as potential contributors to childhood asthma (1). Alongside prospective birth cohort studies, population-level data have shown that a significant decline in childhood antibiotic use is associated with falling asthma incidence in some jurisdictions such as British Columbia (5). This may be relevant to the plateauing of asthma incidence trends in the UK and the US after a decade of rising trends (6–8) and to significant declines in childhood asthma diagnosis in England (9) and Germany (10) in recent years.

Although antibiotics are still among the most commonly used therapeutics for children, concerns about their adverse effects on health have shifted prescription trends in some countries (4). In the Nordic countries, for example, antibiotic prescriptions for children have decreased in the past decade driven by factors such as changed guidelines for otitis media treatment and the introduction of the pneumococcal vaccine (11). Survey data from 132 countries indicated an increase in the proportion of children receiving antibiotics from 36.8% to 43.1% between the years 2005 and 2017 (12). Of these countries, low-income countries showed the greatest relative increase but still showed the lowest rates (39.5%) in 2017. While the incidence of childhood and adult asthma have peaked in many high income jurisdictions, it is still on the rise in many middle and low-income countries (13). Furthermore, asthma incidence in children is still showing a rising trend in new global cohorts of children (14). Although hospital admissions and asthma rates have generally decreased since 2000 (15, 16), these changes are primarily due to shifts in population proportions (14, 17).

Influences on the ecology of the infant gut microbial environment are complex and antibiotics are not the only factor that may play a role in microbial diversity and subsequent atopic disease risk. Other factors associated with childhood asthma include maternal smoking, obesity, infections and antibiotic use during pregnancy, prematurity, caesarean delivery, air pollution, and respiratory infections (16), most of which influence the gut microbiome. A factor of particular interest, breastfeeding, has recently been shown to have protective effects against asthma in children exposed to antibiotics (18) and could mitigate antibiotic-mediated damage to the microbiota through enrichment of *B. longum subsp. Infantis* (19). In high income countries, the proportion of babies having exposure to breastmilk before 6 months has increased between 2000 and 2019. In low income countries, this rate has slightly decreased but exclusive breastfeeding rates have increased (20). Exposure to rich microbiological load in a farm environment has also been observed to decrease allergic outcomes and respiratory disease in children (21). Overall, the *hygiene hypothesis* states that societal changes in developed countries have led to reduced early-life microbial exposures, negatively impacting immunity and increasing the risk of atopic outcomes (2).

Our previous ecological study, conducted between 2000 and 2014 in BC, Canada, revealed an association between antibiotic use before the age of 1 and incidence of asthma diagnosis at 1–4 years old (5). The current study extends the analyses to 2019 at the Local Health Area (LHA) level in BC, aiming to explore if observed associations are the same in recent years and to model the simultaneous effects of changing rates of breastfeeding on asthma incidence.

## Materials and methods

We conducted a population-based ecological study to examine the association between antibiotic use in the first year of life and asthma in children aged 1–4 years of age between 2000 and 2019. The ethics approval for this study was obtained from the University of British Columbia Clinical Research Ethics Board (H09-00650).

### Population-level antibiotic prescribing and asthma incidence

We obtained anonymized antibiotic prescription data from BC PharmaNet; a database that captures information on all outpatient prescriptions dispensed by community pharmacies in the Canadian province of British Columbia (population 5.1 million), except for some drugs used for HIV and STI (22). We calculated the number of prescriptions by age group, year, sex, and local health area (LHA). We obtained population estimates for the 89 LHAs of British Columbia by age group (23). We calculated the mean percentage of children exposed to one or more courses of antibiotics during infancy, mean prescription rates, total number of prescriptions by year, and cumulative percent change in prescription rates over the study duration. We retrieved aggregate data on annual asthma incidence and prevalence from the British Columbia Ministry of Health Chronic Disease Dashboard using a standard case definition for asthma that integrates a combination of diagnostic codes and asthma-specific drug prescription data from BC PharmaNet (24, 25). Particulate matter metrics, indexed to postal codes, and material and social deprivation indices were provided by the Canadian Urban Environmental Health Research Consortium (sourced by the Atmospheric Composition Analysis Group at Dalhousie University, Halifax, Canada) (26, 27).

### Modeling expected fall in asthma incidence due to reduction in antibiotic use and changes in prevalence of breastfeeding using population attributable risk

To model the impact of decreasing exposure to antibiotics in children <1 year and increasing prevalence of breastfeeding on asthma incidence in 1–4 year olds, we calculated the population attributable risk (PAR). PAR is the proportion of disease in a population that would not occur if a risk were removed. When relative risk for an exposure (RR_e_) and the proportion exposed (P_e_) are known, it is derived as follows:

PAR=Pe(RRe−1)/[1+Pe(RRe−1)]

First, we modeled the expected asthma incidence rate (ages 1–4 years) in the BC population in 2019, given the observed reduction in P_e_ (proportion of the infant population <1year of age prescribed one or more courses of antibiotics) under various assumptions for the relative risk associated with antibiotic exposure. Because breastfeeding has been shown to reduce the RR associated with antibiotic exposure, we also modeled a blended relative risk which accounted for the prevalence of breastfeeding. To calculate the change in the proportion of mothers who partially breastfed their last child in BC, we used two different cycles from the Canadian Community Health Survey (CCHS): 2005 and 2017/18 (28). Due to the unavailability of data, the breastfeeding rates for 2000 and 2019 were approximated using CCHS data from 2005 to 2018 respectively, which were the closest years for which data was available. The proportion of breastfeeding outcome from the 2017/18 cycle was determined by Chan et al. (29). The Public Use Microdata Files (PUMFs) were used from the CCHS Maternal Experiences (MEX) module to derive the breastfeeding outcome from the 2005 cycle, available from Statistics Canada through the Abacus Data Network (30). To calculate the proportion of mothers who exclusively or partially breastfed their last child for a duration of at least 6 months, the following equation was used (31): Total weighted number of women 15–55 years giving birth in past 5 years and who breastfed their child ≥6 months/Total weighted number of women 15–55 years giving birth in the past 5 years × 100.

Dai et al. reported adjusted odds ratios of asthma incidence in children who were exposed to antibiotics with breastfeeding (adj. OR of asthma incidence = 1.31) and who were exposed to antibiotics without breastfeeding (adj. OR of asthma incidence = 3.53) in the first year of life (19). Using these estimates, we calculated the antibiotic exposure for each of the breastfeeding exposures considered in this study for calculating the PAR:

RR(abx)=Pe(BF)×1.31+(1−Pe(BF))×3.53

This formula is a weighted average of odds ratios for antibiotic exposure with and without breastfeeding, representing a blended relative risk (RR).

changeinasthmaincidenceattributabletoantibiotics=PAR(2000)×baselineincidence–PAR(2019)×baselineincidence

$$\mathrm{predicted}\;\mathrm{asthma}\;\mathrm{incidence}\;(2019)=\;\mathrm{baseline}\;\mathrm{incidence}\;\text{–}\;\begin{array}{c} \mathrm{change}\;\mathrm{in}\;\mathrm{asthma}\;\mathrm{incidence}\;\\ \mathrm{attributable}\;\mathrm{to}\;\mathrm{antibiotics} \end{array}$$

### Association between antibiotic exposure and asthma incidence using negative binomial modeling

To understand the association between antibiotic exposure in infancy and asthma in early childhood at a finer spatial scale and to account for geographical variability, we examined data segregated according to the 89 LHAs within British Columbia. Negative binomial models were used (as the equidispersion assumption was violated when exploring whether a Poisson regression model could be used). We built a multivariable negative binomial model to estimate the association between the rate of antibiotic prescribing at the LHA-level in infants <1 and asthma incidence at age 1–4 years. The primary independent variable was the antibiotic prescription rate quintile during the first year of life, calculated by categorizing prescription data into five equal-frequency groups based on distribution. The dependent variable was asthma incidence for children aged 1–4 years. Covariates were sex, material and social deprivation indices, and the mean concentration of fine particulate matter [<2.5 μm (PM2.5)] annually in each LHA. We incorporated the LHA as a random effect to account for unmeasured geographical variability. The data was merged from the British Columbia Ministry of Health Chronic Disease Dashboard, BC PharmaNet database, and population estimates data by year, LHA, and sex. Upon observing a changing pattern of antibiotic prescription rates over time, we stratified the data into older years (2000–2014, results of which are published) and recent years (2015–2019) to better capture the differences in the association between asthma incidence and antibiotic prescription quintiles in these periods. We fitted separate negative binomial models for each time period, enabling the analysis of the relationship between asthma incidence and antibiotic prescription quintiles within each timeframe. We performed statistical analysis using R software (version 3.5.2).

## Results

**Figure 1:**
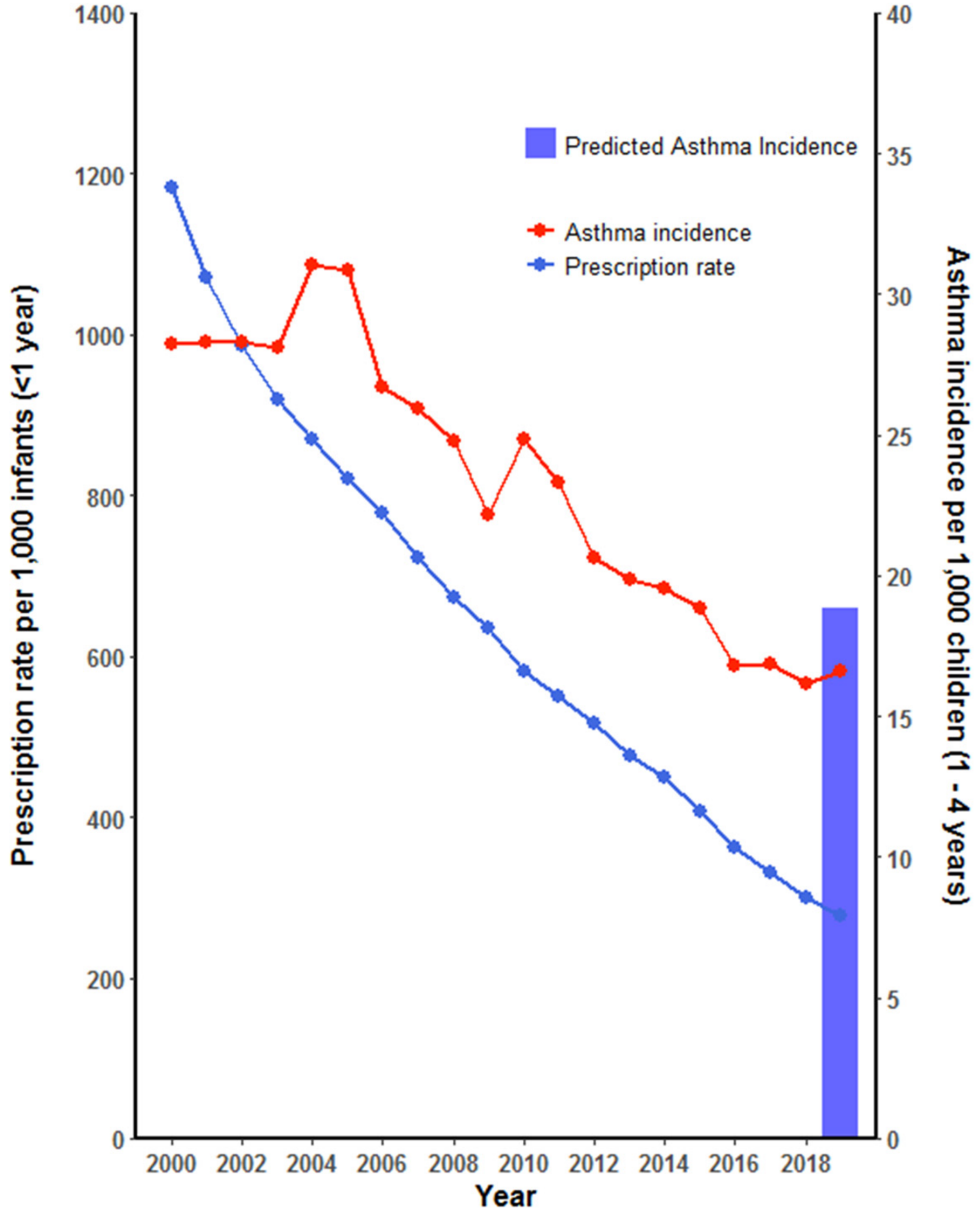
Asthma incidence in BC children aged 1–4 years and 4-years average antibiotic prescription rate during first year of life, 2000–2019. Predicted asthma incidence in BC children using PAR in 2019.

At the population level in British Columbia, from 2000 to 2019, the annual incidence of asthma in children aged 1–4 years showed an absolute decrease of 11.6 new cases per 1,000 children, from 28.3 (27.4, 29.0) in 2000 to 16.6 (16.0, 17.2) per 1,000 children in 2019, a relative decrease of 41% (Figure 1). Between 2000 and 2014, there was an absolute decrease of 8.7 cases per 1,000 children, falling from 28.3 (95% CI 27.5–29.1) cases in 2000 to 19.5 (95% CI 18.9–20.2) cases in 2014, a relative decrease of 31%. However, after 2014, the rate of decrease in asthma incidence in children appears to be stabilising.

**Figure 2:**
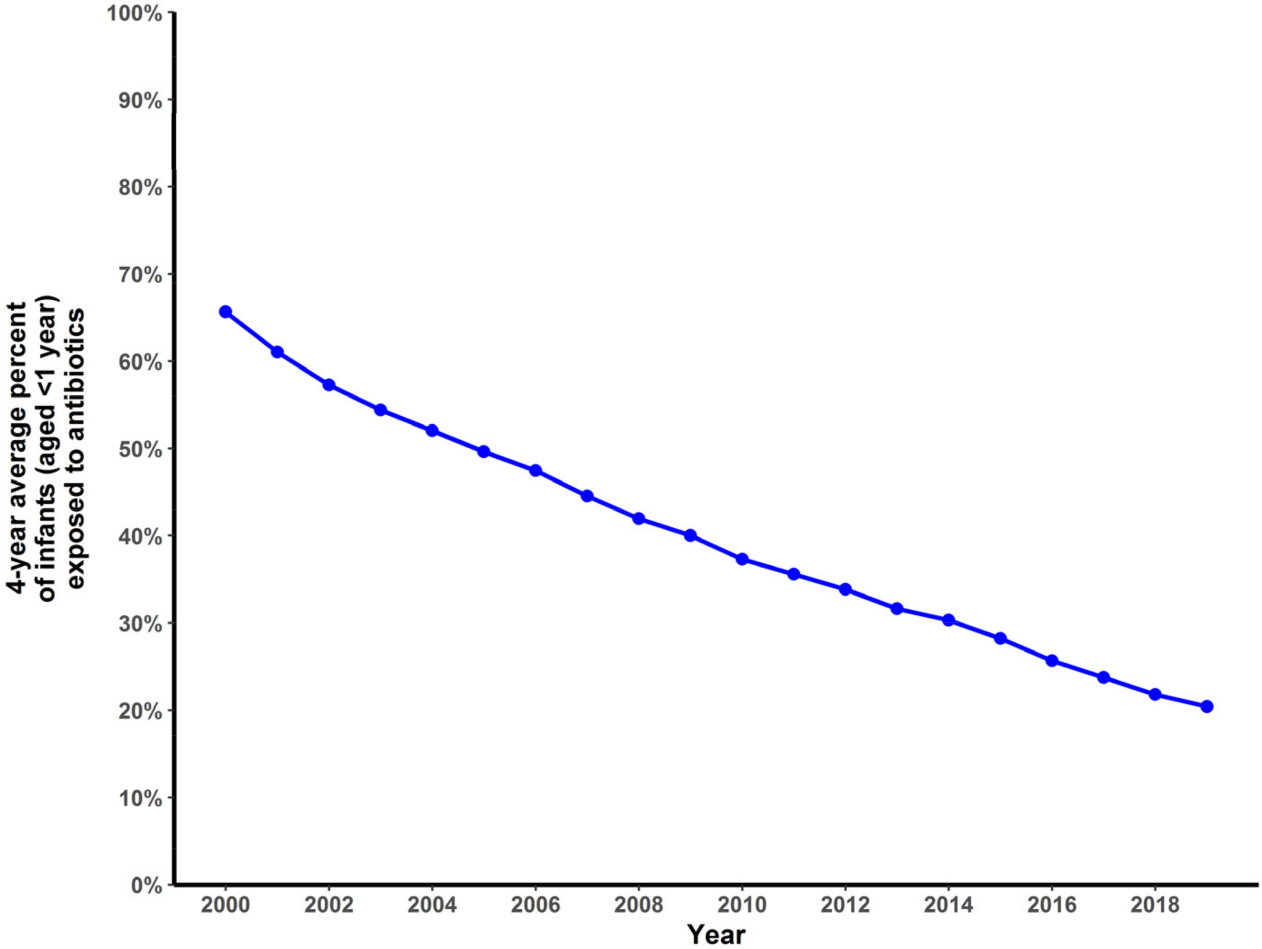
Four-year average percentage of BC infants (<1 year) who received one or more antibiotic prescriptions in their first year of life, 2000–2019.

**Figure 3:**
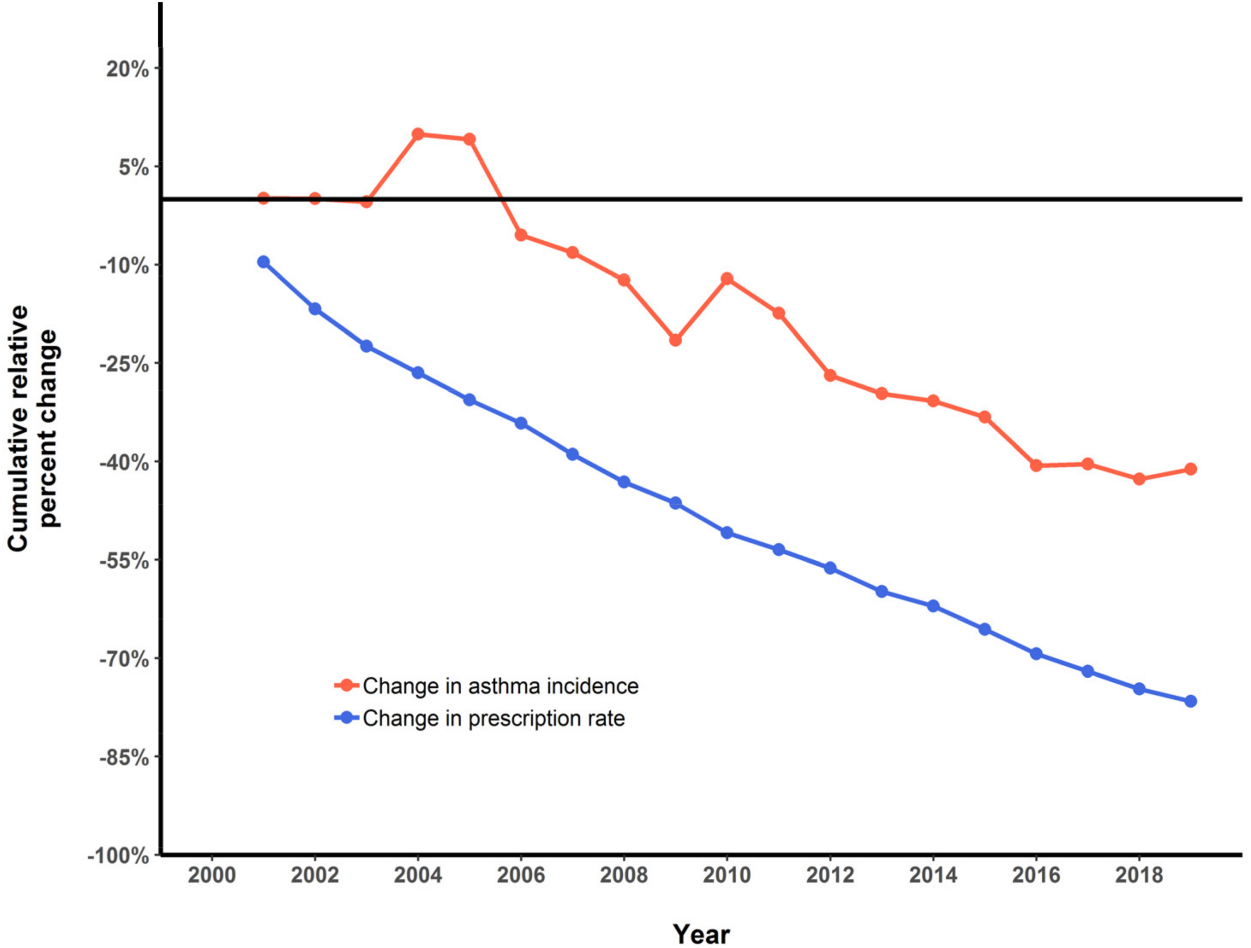
Cumulative relative percent change in 4-year average antibiotic prescription rate (<1 year of age) and asthma incidence (1–4 year of age) in BC children, 2000–2019.

In 2000, for children 1–4 years of age, the corresponding 4-year mean annual antibiotic prescription for infants (aged <1 year) was 1,183.7 per 1,000 infants. This rate decreased to 276.74 in 2019, an absolute decrease of 906.96 per 1,000 infants and a relative decrease of 77% (Figure 1). The average proportion of infants exposed to one or more courses of antibiotics before the age of 1 year decreased by 46%, from 66% in 2000 to 20% in 2019 (Figure 2), representing a cumulative relative decrease in annual antibiotic prescription rate of 76% (Figure 3). The average proportion of infants exposed to one or more courses of antibiotics before the age of 1 year decreased from 30% in 2014 to 20% in 2019 (Figure 2). While we observed a decrease in asthma incidence during the study period, we also observed a decrease in asthma prevalence during the same period (Supplementary Figure 1). However, similar to the incidence of asthma, the prevalence of asthma has also stabilised during the most recent years of the study. Asthma incidence in children (1–4 years of age) and antibiotic use in the first year of life were strongly correlated (Spearman's *r* = 0.9489, *p* < 0.001). Amoxicillin was the most frequently prescribed antibiotic for infants across all years of the study, making up 6,916 (68·5%) of 10,096 antibiotic prescriptions in 2019 (Supplementary Table 1).

### Predicting asthma incidence with various levels of exposure to antibiotics and breastfeeding

Following the population attributable risk calculation and using a range of hypothetical relative risk scores, we modelled expected asthma incidence after observing the decrease in antibiotic exposure. With the reduction in antibiotic exposure and a relative risk (RR) of asthma of 2, we would predict an annual asthma incidence decrease between 2000 and 2019 from 28.3 per 1,000 children to 22.3 per 1,000 children. With a relative risk of asthma to be 3, we would predict an annual asthma incidence decrease between 2000 and 2019 from 28.3 per 1,000 children to 21.0 per 1,000 children. However, the actual observed asthma incidence in this study decreased to 16.6 per 1,000 children in 2019 (Figure 1).

In our final model to predict asthma incidence with the antibiotic exposure reduction, we included the modelled changes in breastmilk exposure. Using the equation outlined in the methods, the proportion of BC mothers who exclusively or partially breastfed their child for a duration of at least 6 months in the 2005 CCHS cycle was 386/831 × 100 = 46%. And in the 2017/18 cycle of the CCHS, Chan et al. calculated 70.7% of respondents in BC reported breastfeeding their child born in the last 5 years (either exclusively or partially) for 6 months or more (29). We calculated the relative risk of asthma incidence associated with antibiotic exposure and the corresponding breastfeeding exposure using the method as described in the methods section. For breastfeeding exposure of 46% in 2005 (as a proxy for exposure in 2000) and the antibiotic exposure observed in 2000, the blended relative risk of asthma incidence was calculated as 2.53 (Table 1). For breastfeeding exposure of 71% in 2018 (as a proxy for exposure in 2019) and the antibiotic exposure observed in 2019, the blended relative risk of asthma incidence was calculated as 1.98. Using a decreased relative risk of asthma incidence from 2.53 in 2000 to 1.98 in 2019 and an increase in breastfeeding exposure from 0.46 in 2005 to 0.71 in 2018, we predicted the annual asthma incidence to decrease from 28.3 per 1,000 children in 2000 to 18.8 per 1,000 children in 2019. The above predicted annual asthma incidence was very close to the observed annual asthma incidence (16.6 per 1,000 children in 2019) when incorporating changes in both antibiotic and breastmilk exposures (Figure 1).

**Table 1:** Counterfactual model to demonstrate the effect of adding breastfeeding to the PAR model.

Scenario	P(antibiotics)	P(breastfed)	Blended RR	Asthma incidence
2000 baseline (observed)	66%	46%	2.53	28.3/1,000 pop
2019 counterfactual model	20%	46%	2.53	22.2/1,000 pop
2019 model (observed)	20%	71%	1.98	18.8/1,000 pop

### Association between antibiotic exposure and asthma incidence

**Figure 4:**
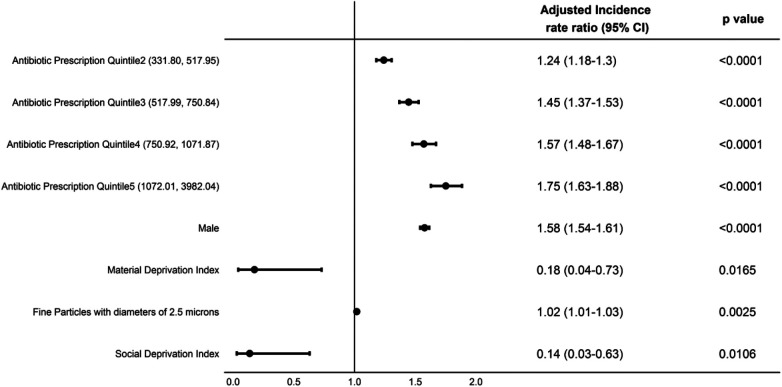
Adjusted incidence rate ratios (aIRRs) predicting asthma incidence in BC children aged 1–4 years, 2000–2014.

**Figure 5:**
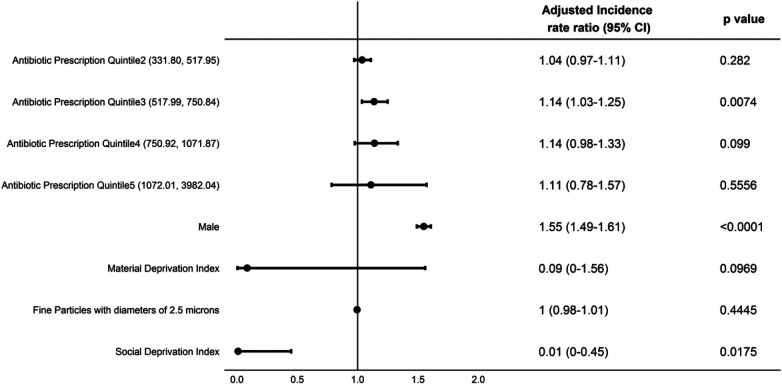
Adjusted incidence rate ratios (aIRRs) predicting asthma incidence in BC children aged 1–4 years, 2015–2019.

Over the study period both the minimum and maximum average annual prescription rate per 1,000 children per year decreased, mostly observed during the latter years (Table 2). Using the negative binomial model, we calculated adjusted incidence rate ratios (aIRRs) predicting asthma incidence and observed a significant association between the quintiles of average annual antibiotic prescription rate and asthma incidence for the period from 2000 to 2014. Expected incidence of asthma for children under Quintile 5 (antibiotic prescription range 1,072.01–3,982.04 per 1,000 infants) was estimated to be 75% (aIRR 1.75, 95% CI: 1.63–1.88, *P* < 0.0001) higher than that for Quintile 1 (antibiotic prescription range 30.97–331.67 per 1,000 infants) (Figure 4). We observed a dose-response relationship when looking at asthma risk for the children under other Quintiles of prescribing compared to Quintile 1. However, during the period of 2015–2019, this association appeared to be weakened, with the expected asthma incidence for Quintile 5 only 11% (aIRR 1.11, 95% CI: 0.78–1.57) higher than for Quintile 1 (Figure 5), and this difference was not statistically significant. Moreover, we didn't observe a dose-response relationship for asthma risk as observed for the duration of 2000–2014. We initially hypothesized that the observed weakening association between antibiotic prescription rates and asthma incidence in recent years (2015–2019) might be attributed to a reduction in the highest rates of antibiotic prescriptions, particularly within Quintile 5. To test this hypothesis, we performed additional analyses controlling for extreme antibiotic prescription rates. Even after excluding prescription rates above a threshold of 2,881 per 1,000 infants (which represents the maximum prescription rate in the recent years, 2015–2019) (Table 2), the association between antibiotic prescription rates and asthma incidence in the older years (2000–2014) remained stronger than in the recent years (Data not shown). We found that boys had more than 50% higher risk of asthma in both time periods of this study (Figures 4, 5).

**Table 2:** Four-year average prescription rate ranges of infants (<1 year) in BC, 2000–2019.

Year	Min	Max
2000	387.9	3,817.3
2001	327.5	3,696.2
2002	280.0	3,914.9
2003	187.3	3,982.0
2004	143.2	3,931.6
2005	161.7	3,790.1
2006	148.3	3,552.2
2007	128.3	3,184.0
2008	172.1	3,061.5
2009	154.2	2,864.2
2010	185.5	2,678.0
2011	135.7	2,833.9
2012	102.4	2,565.1
2013	130.4	2,646.6
2014	140.5	2,901.7
2015	111.5	2,881.1
2016	135.7	2,822.7
2017	115.9	2,240.8
2018	86.5	1,840.0
2019	31.0	1,781.3

## Discussion

In our population-based study of 5.1 million people in British Columbia, we noticed a 41% decrease in asthma incidence between 2000 and 2019 in children 1–4 years of age, which correlated with a 77% decrease in the corresponding antibiotic prescription rate in the first year of life. The observed time gap between the decrease in antibiotic exposure and decrease in asthma incidence is likely reflecting the 4 years required to gather data on a full cohort of children 1–4 years of age. Patrick et al. reported a 26% decrease in asthma incidence between 2000 and 2014 in a similar study in BC, Canada (5). Our study extended the study period and explored if the previously observed decline in asthma incidence in BC is consistent during the recent years (2015–2019). We observed that asthma incidence has stabilised between 2015 and 2019 with the exposure to one or more courses of antibiotics before the age of 1 year having decreased from 30% in 2014 to 20% in 2019. We also found a similar trend when looking at the asthma prevalence in BC for the same duration. However, there was a strong correlation between asthma incidence in children aged 1–4 years and antibiotic exposure during the first year of life.

In addition to a decrease in antibiotic exposure, other factors such as reductions in air pollution and an increase in breastfeeding exposure are likely to have contributed to the observed associations (5, 32). In this study, we modelled the expected incidence of asthma with a range of relative risk estimates. This method assumed the decrease in antibiotic exposure in infancy and increase in breastfeeding exposure were major independent predictors of asthma incidence at the population level. We couldn't explain most of the observed decrease in asthma incidence in this study, when the model included antibiotic exposure only. However, when modelled using both the observed decrease in antibiotic exposure and available data on breastfeeding exposure, we predicted the asthma incidence of 18.8 per 1,000 children in 2019, which is very close to the observed rate (16.6 per 1,000 children in 2019). Therefore, higher rates of breastfeeding in BC in recent years is likely another contributing factor to why the true asthma incidence rate was lower than the predicted asthma incidence when only antibiotic prescribing was considered. We used partial breastfeeding exposure without accounting for breastfeeding duration. Previously, Patrick et al. reported no significant association between breastfeeding duration and asthma risk. However, authors hypothesized that breastfeeding could act as a effect modulator and may regulate the gut during antibiotic disruption, restoring bacterial diversity and largely mitigating the risk of asthma (5). Moreover, Dai et al. reported clear protection from the risk of developing asthma when children are breastfed while exposed to antibiotics (19).

In our study, we calculated an aIRR predicting asthma incidence, and observed a significant association between the quintiles of average annual antibiotic prescription rate in the first year of life and asthma incidence in children 1–4 years of age. However, when the aIRRs was calculated for 2000–2014 and 2015–2019 separately, we observed that the aIRR is weaker compared to that during 2000–2014. A lower relative risk associated with antibiotics is what we would predict in a population with more exposure to breast feeding. This finding along with the modelled expected asthma incidence may suggest that decreases in antibiotic exposure may not be the only major factor in decreasing asthma risk and that there are other factors playing a role in reducing asthma risk, such as breastfeeding. Dai et al. reported that compared with children with no antibiotic exposure in the first year of life, antibiotic-exposed children who were not breastfed had 3-fold higher odds of developing asthma, whereas there was no such association in antibiotic-exposed children who were breastfed (19).

## Limitations

This ecological study has several limitations. Due to unavailability, we were not able to use the breastfeeding exposure data for the same year as for the antibiotic exposure and the breastfeeding data that we used for predicting the asthma incidence was based on the Canadian Community Health Survey which has its own limitations. The actual breastfeeding exposure might be different during the years for which antibiotic exposures were included in the model of expected asthma risk. Moreover, we couldn't analyze the effect of various forms of breastfeeding such as exclusive or partial breastfeeding or pumped breast milk. We couldn't analyze the effect of individual antibiotic classes on asthma risk and our data didn't allow us to account for indications given for antibiotic use. For the asthma diagnoses, we relied on the BC chronic disease registry database which rely on clinical diagnostic codes which may lead to some misclassification. This study was designed to address some of the gaps identified in our previous ecological study and to observe if the previous finding is still consistent with the more recent years of data. However, there are many details or gaps which are not possible to address through the ecological analysis. Patient-level factors that were outlined in the introduction cannot be adjusted for using this design, of particular importance being the indication for antibiotic prescribing, and therefore inferences cannot be drawn at the patient-level. In recognition of this limitation, and to build upon this work, we have designed a population-based cohort study comprising all infants born in BC between 2001 and 2011, following up to age 7, with the aim of evaluating the effect of reducing antibiotic use on asthma, allergic rhinitis and atopic dermatitis (33). This will provide an opportunity to account for variations at the individual-level and offer insights into subpopulations receiving antibiotics for different indications to thoroughly investigate and adjust for potential confounding by indication. It will also allow for an investigation into whether different types of antibiotics confer differential risk of atopic outcomes.

## Conclusion

We identified that over the past 20 years antibiotic exposure in infancy has decreased significantly in BC, as has asthma incidence in children 1–4 years of age, with asthma incidence stabilising in more recent years. Asthma risk might not be explained by only antibiotic exposure. Air pollution and other social and economic factors as well as breastfeeding during early childhood are important factors to account for when analysing asthma risk in children.

## Data Availability

The data analyzed in this study is subject to the following licenses restrictions: access to data provided by the Data Stewards is subject to approval but can be requested for research projects through the Data Stewards or their designated service providers. Requests to access these datasets should be directed to https://healthdataplatformbc.ca. The following data sets were used in this study: Chronic Disease Registry and Pharmanet. All inferences, opinions, and conclusions drawn in this publication are those of the author(s), and do not reflect the opinions or policies of the Data Steward(s). The following BC Ministry of Health datasets were used in this study: Chronic Disease Registry and Pharmanet. All inferences, opinions, and conclusions drawn in this publication are those of the author(s), and do not reflect the opinions or policies of the Data Steward(s).
