# Supplementary material for: Association between antibiotic usage during infancy and asthma incidence among children: a population-level ecological study in British Columbia, Canada
Source: Front Allergy. 2024 Aug 27;5:1456077. doi: 10.3389/falgy.2024.1456077 (PMC11403638; doi:10.3389/falgy.2024.1456077)
Supplement: Supplementary file 1 [file Datasheet1.docx]

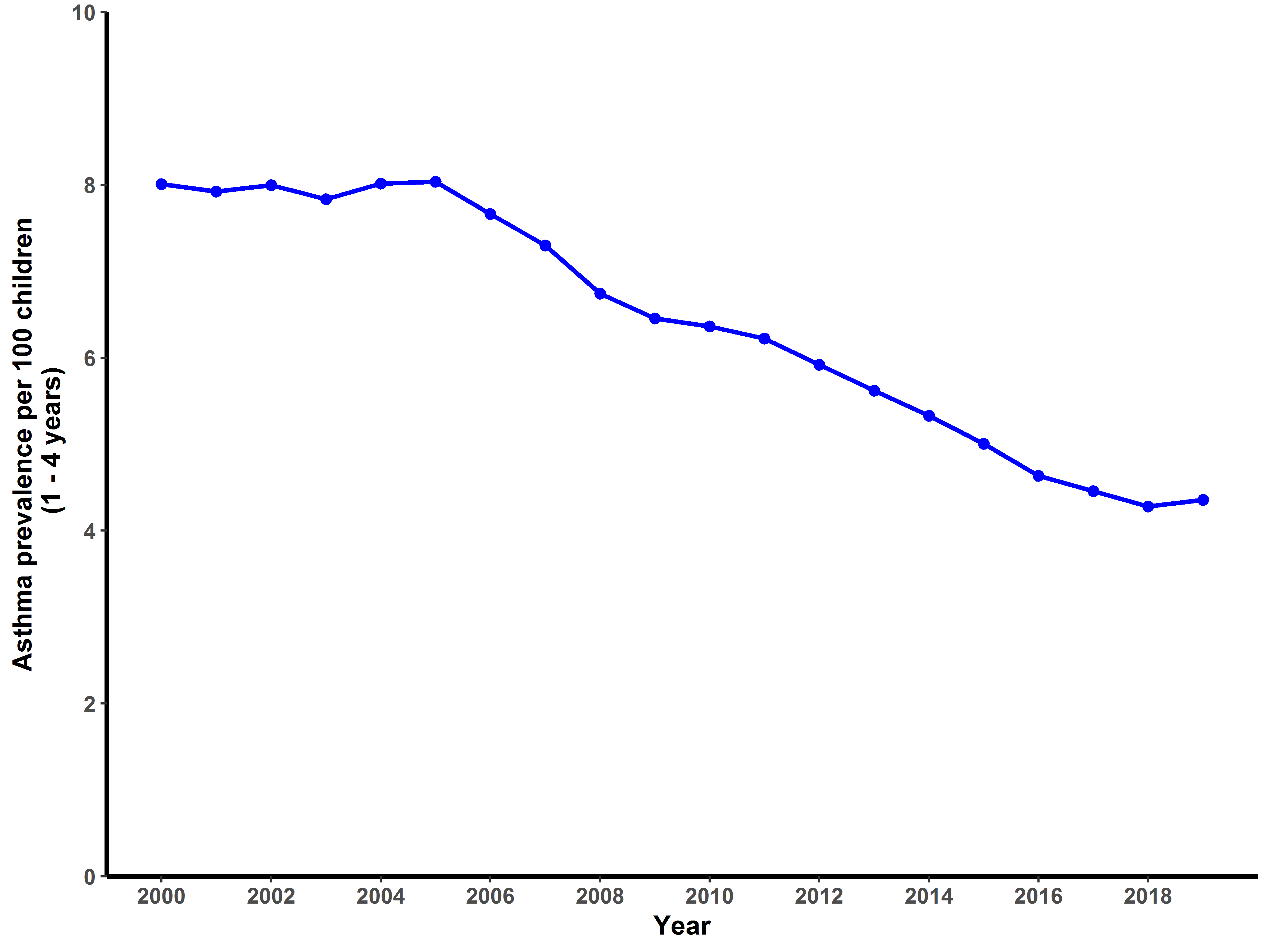


**Fig S1**: Asthma prevalence among BC children of 1-4 years of age, 2000-2019

**Table S1**: Most common antibiotics prescribed to children under age 1 year in British Columbia, 1996-2019

| year | Amoxicillin (%) | Sulfamethoxazole and trimethoprim (%) | Cefaclor (%) | Erythromycin (%) | Cefalexin (%) | Amoxicillin and enzyme inhibitor (%) | Cefixime (%) | Clarithromycin (%) | Cloxacillin (%) | Azithromycin (%) | Total number of prescriptions |
| --- | --- | --- | --- | --- | --- | --- | --- | --- | --- | --- | --- |
| **1996** | 60.670 | 13.790 | 8.980 | 7.200 | 2.680 | 2.010 | 1.930 | 1.460 | 1.260 | 0.020 | 60611 |
| **1997** | 61.890 | 13.230 | 8.310 | 7.020 | 3.300 | 1.650 | 1.770 | 1.330 | 1.270 | 0.220 | 51064 |
| **1998** | 63.250 | 13.030 | 7.100 | 6.290 | 3.550 | 1.790 | 1.600 | 1.750 | 1.180 | 0.460 | 45089 |
| **1999** | 63.390 | 12.510 | 6.390 | 5.440 | 3.970 | 1.850 | 1.650 | 2.840 | 1.090 | 0.880 | 39492 |
| **2000** | 61.730 | 11.530 | 5.110 | 5.640 | 4.380 | 1.990 | 1.740 | 5.010 | 0.930 | 1.930 | 36166 |
| **2001** | 61.650 | 10.720 | 4.630 | 4.200 | 5.210 | 1.890 | 2.070 | 6.350 | 0.780 | 2.510 | 33801 |
| **2002** | 60.900 | 9.480 | 4.090 | 3.250 | 5.260 | 1.960 | 2.140 | 7.640 | 0.800 | 4.490 | 32427 |
| **2003** | 58.960 | 8.620 | 3.780 | 2.870 | 5.860 | 2.400 | 2.000 | 8.380 | 0.780 | 6.350 | 31318 |
| **2004** | 58.550 | 8.680 | 3.440 | 1.980 | 6.410 | 2.140 | 1.730 | 8.680 | 0.840 | 7.560 | 28344 |
| **2005** | 58.670 | 7.970 | 3.110 | 1.920 | 6.660 | 2.410 | 2.040 | 8.670 | 0.780 | 7.790 | 27602 |
| **2006** | 59.050 | 8.350 | 2.630 | 1.510 | 6.240 | 2.180 | 2.150 | 8.640 | 0.860 | 8.390 | 25007 |
| **2007** | 59.950 | 8.510 | 2.160 | 1.250 | 6.410 | 2.280 | 2.150 | 8.380 | 0.750 | 8.150 | 24905 |
| **2008** | 60.830 | 7.180 | 2.050 | 1.360 | 6.890 | 2.060 | 2.550 | 8.890 | 0.730 | 7.470 | 24917 |
| **2009** | 61.850 | 7.010 | 1.600 | 1.020 | 7.330 | 2.120 | 2.700 | 8.500 | 0.750 | 7.120 | 21411 |
| **2010** | 63.860 | 6.330 | 0.340 | 0.680 | 6.850 | 2.070 | 3.110 | 9.600 | 0.720 | 6.430 | 21772 |
| **2011** | 65.760 | 3.720 | 0.000 | 0.260 | 7.510 | 3.030 | 3.180 | 9.630 | 0.630 | 6.270 | 20205 |
| **2012** | 64.250 | 3.010 | 0.110 | 0.030 | 7.890 | 2.970 | 3.770 | 9.710 | 0.400 | 7.840 | 17657 |
| **2013** | 63.540 | 4.980 | 0.060 | 0.010 | 8.700 | 3.700 | 4.150 | 7.520 | 0.010 | 7.340 | 16882 |
| **2014** | 67.510 | 2.780 | 0.050 | 0.000 | 9.210 | 3.960 | 3.000 | 6.030 | 0.140 | 7.330 | 14630 |
| **2015** | 65.780 | 4.990 | 0.040 | 0.140 | 9.980 | 4.760 | 0.090 | 5.420 | 0.320 | 8.480 | 13182 |
| **2016** | 67.270 | 5.310 | 0.000 | 0.170 | 10.230 | 3.100 | 1.120 | 4.490 | 0.280 | 8.040 | 12684 |
| **2017** | 67.310 | 3.670 | 0.000 | 0.090 | 10.570 | 4.060 | 2.850 | 3.580 | 0.270 | 7.590 | 11661 |
| **2018** | 69.830 | 0.440 | 0.000 | 0.000 | 11.260 | 4.520 | 3.750 | 2.870 | 0.230 | 7.110 | 10918 |
| **2019** | 68.480 | 0.100 | 0.000 | 0.000 | 12.420 | 4.680 | 4.490 | 2.000 | 0.260 | 7.580 | 10096 |
| **2020** | 60.320 | 0.190 | 0.000 | 0.000 | 19.070 | 5.850 | 6.920 | 1.710 | 0.440 | 5.500 | 5741 |
